# A humanized 4-1BB-targeting agonistic antibody exerts potent antitumor activity in colorectal cancer without systemic toxicity

**DOI:** 10.1186/s12967-022-03619-w

**Published:** 2022-09-08

**Authors:** Lian-sheng Cheng, Yong-feng Cheng, Wen-ting Liu, Aolin Shen, Dayan Zhang, Tingjuan Xu, Wu Yin, Min Cheng, Xiaopeng Ma, Fengrong Wang, Qun Zhao, Xiaoli Zeng, Yan Zhang, Guodong Shen

**Affiliations:** 1 Department of Geriatrics, The First Affiliated Hospital of University of Science and Technology of China, Gerontology Institute of Anhui Province, Division of Life Sciences and Medicine, University of Science and Technology of China, Hefei, 230001 Anhui China; 2 Hefei HankeMab Biotechnology Limited, Hefei, 230088 Anhui China; 3 Anhui Provincial Key Laboratory of Tumor Immunotherapy and Nutrition Therapy, Hefei, 230001 Anhui China; 4 Department of Genetics, School of Life Science, Anhui Medical University, Hefei, 230032 Anhui China; 5 Department of General Surgery, The First Affiliated Hospital of Anhui Medical University, Hefei, 230032 Anhui China; 6 Department of Thyroid and Breast Surgery, The First Affiliated Hospital of University of Science and Technology of China, Division of Life Sciences and Medicine, University of Science and Technology of China, Hefei, 230001 Anhui China; 7 School of Health Service Management, Anhui Medical University, Hefei, 230032 Anhui China

**Keywords:** 4-1BB/CD137, Monoclonal antibody, Targeted therapy, Cancer immunotherapy, Antitumor immunity, Toxicity

## Abstract

**Background:**

Colorectal cancer (CRC) is one of the most common malignancies and the patient survival rate remains unacceptably low. The anti-programmed cell death-1 (PD-1)/programmed cell death ligand 1 (PD-L1) antibody-based immune checkpoint inhibitors have been added to CRC treatment regimens, however, only a fraction of patients benefits. As an important co-stimulatory molecule, 4-1BB/CD137 is mainly expressed on the surface of immune cells including T and natural killer (NK) cells. Several agonistic molecules targeting 4-1BB have been clinically unsuccessful due to systemic toxicity or weak antitumor effects. We generated a humanized anti-4-1BB IgG4 antibody, HuB6, directed against a unique epitope and hypothesized that it would promote antitumor immunity with high safety.

**Methods:**

The antigen binding specificity, affinity and activity of HuB6 were determined by enzyme-linked immunosorbent assay (ELISA), surface plasmon resonance (SPR), biolayer interferometry (BLI) and flow cytometry. The antitumor effects were evaluated in humanized mice bearing syngeneic tumors, and possible toxicity was evaluated in humanized mice and cynomolgus monkeys.

**Results:**

HuB6 showed high specificity and affinity for a binding epitope distinct from those of other known 4-1BB agonists, including utomilumab and urelumab, and induced CD8 + T, CD4 + T and NK cell stimulation dependent on Fcγ receptor (FcγR) crosslinking. HuB6 inhibited CRC tumor growth in a dose-dependent manner, and the antitumor effect was similar with urelumab and utomilumab in humanized mouse models of syngeneic CRC. Furthermore, HuB6 combined with an anti-PD-L1 antibody significantly inhibited CRC growth in vivo. Additionally, HuB6 induced antitumor immune memory in tumor model mice rechallenged with 4 × 10^6^ tumor cells. Toxicology data for humanized 4-1BB mice and cynomolgus monkeys showed that HuB6 could be tolerated up to a 180 mg/kg dose without systemic toxicity.

**Conclusions:**

This study demonstrated that HuB6 should be a suitable candidate for further clinical development and a potential agent for CRC immunotherapy.

**Supplementary Information:**

The online version contains supplementary material available at 10.1186/s12967-022-03619-w.

## Background

Colorectal cancer (CRC) is one of the most common malignancies, and the patient survival rate remains unacceptably low [1]. Recently, monoclonal antibody (mAb)-based immune checkpoint inhibitors, particularly anti-programmed cell death-1 (PD-1)/programmed cell death ligand 1 (PD-L1) mAbs, have been added to CRC treatment regimens [2]. However, only a fraction of patients benefits from the therapy [3, 4].

4-1BB, also called CD137 or tumor necrosis factor receptor superfamily member 9 (TNFRSF9), is a costimulatory molecule expressed functionally on the surface of various types of leukocytes, such as T cells, natural killer (NK) cells and subsets of dendritic cells, and can be activated by its ligand 4-1BBL or activating anti-4-1BB antibodies to enhance tumor rejection; thus, it is regarded as a potential target for cancer immunotherapy [5–7].

Several anti-4-1BB agonistic antibodies have advanced to clinical stages but have never been clinically successful because of the intolerable toxicity caused by systemic immune activation [8]. Urelumab (BMS-663513), an IgG4 mAb, caused severe hepatotoxicity in more than 5% of patients enrolled in phase I and II clinical trials [9]. In contrast, utomilumab (PF-05082566), an IgG2 mAb, showed fewer grade III–IV adverse effects and no dose-limiting toxicity up to the highest dose of 10 mg/kg, but it produced a much milder agonistic function than urelumab [10, 11]. Therefore, new antibody drugs that effectively and safely target 4-1BB are urgently needed.

Here, we demonstrate that HuB6, a novel human recombinant anti-4-1BB mAb with high specificity, has a binding epitope distinct from those of other known anti-4-1BB mAbs and shows potent antitumor activity and immune memory induction in humanized mouse models bearing CRC tumors and no systemic toxicity in either humanized mice or cynomolgus monkeys.

## Methods

### Cell culture

CHO-K1 and HEK-293 cells were obtained from American Type Culture Collection (ATCC CCL-61 and CRL-1573). HEK-293/NFκB-Luci/4-1BB cells were genetically engineered and expressed human 4-1BB and a luciferase reporter driven by a response element sensitive to 4-1BB agonistic stimulation and cultured in DMEM supplemented with 1 μg/mL puromycin (Gibco, C11995500BT) and 800 μg/mL hygromycin B (Sangon Biotech, A600230-0001). CHO-K1/CD32A, CHO-K1/CD32B, CHO-K1/CD16 and CHO-K1/hu4-1BB cells were designed to express the human Fcγ receptors (FcγRIIA, FcγRIIB, FcγIRA) and 4-1BB on the cell membrane, respectively, and grown in DMEM/F12 (HyClone, SH30023.01) containing 1 mg/mL Geneticin (Gibco, 11811023). The murine and human CRC cell lines CT26 and Colo205 were obtained from the cell bank affiliated with the Shanghai Institute of Biochemistry and Cell Biology (SIBCB), and the murine CRC cell line MC38 was purchased from Cobioer Company (Nanjing, China), authenticated, tested for mycoplasma contamination and cultured in RPMI-1640 medium (HyClone, SH30809.01). All media were supplemented with 10% fetal bovine serum (Ausbian, VS500T) and a 1% penicillin–streptomycin solution (HyClone, SV30010), and cells were cultured at 37 °C in a humidified incubator with 5% CO2.

### Protein expression and purification

The monomeric antigen (mono-Hu4-1BB) was produced by introducing a mutation (C121S) with His-tag at the C terminus. The sequences of urelumab and utomilumab were individually obtained from the patents US8137667B2 and US2012/0237498A1 and that of anti-CD3 antibody (clone: OKT3, IMGT/mAb-DB, ID: 92) was obtained from IMGT/mAb-DB (http://www.imgt.org/mAb-DB). The sequences for 4-1BB of mouse and cynomolgus monkey and human 4-1BBL were obtained from UniProt (mouse 4-1BB: P20334, cynomolgus monkey 4-1BB: A9YYE7, human 4-1BBL: P41273). The antigens and 4-1BBL were generated by cloning DNA-encoding sequences with a mouse Fc tag sequence at the C terminus independently into the multiple cloning sites of the mammalian expression vector pcDNA 3.4 TOPO (Invitrogen, A14697). Transfection was conducted with Expi293F cells (Gibco, A14635) and the cell culture was collected in 96 h. The Antigens with mouse Fc tag and antibodies were purified through 1 mL MabSelect PrismA column (GE Healthcare) and the mono-Hu4-1BB was directly purified on a HisTrap excel nickel column (GE Healthcare) according to a previously published protocol [10]. In addition, human IgG, used as an isotype control, was purchased from GenScript Biotech Corporation (Nanjing, China).

### Enzyme linked immunosorbent assay (ELISA)

The indirect ELISA method was used, and plates were coated with 4-1BB-ECD (extracellular domain)-mFc (6 nM 4-1BB-ECD-mFc, 1 μg/mL mouse 4-1BB-ECD-mFc or 350 ng/mL cynomolgus 4-1BB-mFc) in carbonate buffer at 4 °C overnight. After blocking with 1% BSA at 37 °C for 2 h, serially diluted test antibodies were added, incubated at room temperature for 2 h and then subjected to detection with HRP-conjugated secondary antibodies (goat anti-human Fc, Jackson ImmunoResearch Laboratories, 146460). After washing with a PBST solution three times, TMB (Invitrogen, 002023) was added as a substrate, and the absorbance was detected at 405 nm. Meanwhile, this ELISA protocol was also used to analyze the binding of HuB6 to other human TNF receptor superfamily (TNFRSF) proteins (CD40: CD4-HM140; OX40: P43489, CD27: CD2-HM127) purchased from Kactus Biosystems (Shanghai, China).

### Antibody affinity determination

The affinity of HuB6, utomilumab or urelumab for human 4-1BB was determined at 25 °C by surface plasmon resonance (SPR) using a Biacore T200 carried out in single-cycle mode with a protein A biosensor chip (GE Healthcare) and biolayer interferometry (BLI) using Octet Red96 system (Pall ForteBio Analytics) according to the manufacturers’ manuals. Briefly, for SPR measurement, human 4-1BB ECD was coupled on a Series S Sensor Chip CM5 (29104988, GE Healthcare) to 300 RU using an Amine Coupling Kit (BR100050, GE Healthcare). The tested antibody and control (80 μg/mL) with two-fold serial dilutions were injected across the immobilized human 4-1BB surface at a flow rate of 30 μL/min (association and dissociation time, 300 s, stabilization time, 120 s). The chip surface was regenerated by an injection of 50 mM NaOH at a flow rate of 30 μL/min for 60 s at the end of each cycle. Binding of antibodies to human 4-1BB was analyzed using a 1:1 Langmuir model. The kinetic rate constants, association rate constant (Kon), dissociation rate constant (Koff) and equilibrium dissociation constant (KD), were calculated using the evaluation software (version 3.1, GE Healthcare). For the BLI method, the antibody was prepared at 5, 10 and 20 μg/mL in 1 × PBS running buffer and dispensed into a 96-well tilted-bottom microplate and a second 96-well microplate contained human 4-1BB (Hu4-1BB-His, Acro biosystems) at the seven titrated concentrations (200–12.5 nM with two-fold dilutions). Antibodies were loaded onto AHC biosensor for a 200-s loading step. After a 60-s baseline dip in 1 × PBS buffer, the binding kinetics were measured by dipping the antibody-coated sensors into the wells containing human 4-1BB. The binding interactions were monitored over a 500-s association period followed by a 30-min dissociation period in new wells containing fresh 1 × PBS buffer. Dissociation constants were calculated from raw data with analysis software (version 6.3, ForteBio).

### Competitive protein binding assay

CHO-K1-Hu4-1BB cells were cultured to 80% confluence, digested with trypsin, centrifuged at 1000 rpm for 5 min and collected in EP tubes. HuB6, utomilumab and urelumab were labeled with biotin to create the corresponding bio-antibodies and diluted to 1.5 μg/mL with PBS. The working concentration of human 4-1BBL ranged from 90 to 0.35 μg/mL with a fourfold gradient dilution. Each Bio-antibody was mixed with 4-1BBL and then incubated with CHO-K1-Hu4-1BB cells for 30 min. After washing twice with a PBS buffer solution, the cells were incubated with a streptavidin-FITC secondary antibody (BioLegend, 405202) and incubated for 30 min in the dark. Finally, the prepared cells were suspended in 500 μL PBS and detected by flow cytometry (Beckman Coulter, CytoFLEX). In addition, the flow cytometry analysis protocol was used to evaluate the selectivity of HuB6 between 4-1BB and the other TNFRSF proteins including OX40, CD40 and CD27, which were transiently overexpressed on the surface of HEK293F cells. The goat anti-human Fc-PE (14–4998-82, Invitrogen) was used as the secondary antibody.

### Luciferase assay

The activity of the mAb HuB6 was detected using the luciferase reporter gene method in vitro. The same number (3 × 10^4^)of HEK-293/NFκB-Luci/4-1BB cells and CHO-K1 cells expressing different FcγRs per well were seeded in 96-well plates and incubated with serially diluted antibodies overnight in a CO2 incubator at 37 °C.Then, both firefly luciferase activity and Renilla luciferase activity were measured with a Glomax multidetection system luminometer using a Dual-Luciferase Reporter Assay System (Promega). Firefly luciferase activity was normalized against Renilla luciferase activity to measure antibody activity.

### Mutant antigen binding detection

Extracellular amino acid sites (M101, I32, and N42) in the human 4-1BB antigen were independently mutated to synthesize different target genes, which were inserted into the pcDNA3.4 vector to obtain DNA plasmids carrying the different 4-1BB antigens. Expi293F cells expressing mutant or wild-type (WT) 4-1BB antigens on the surface were obtained by transient transfection and incubated with utomilumab, urelumab or HuB6 at a concentration of 10 μg/mL and subsequent threefold gradient dilution for 30 min. After washing twice with PBS buffer, the cells were incubated with a FITC-conjugated goat anti-human IgG (H + L) secondary antibody (Invitrogen, H10301) for 30 min, washed and resuspended in PBS, and then subjected to an antibody binding assay evaluated by flow cytometry (Beckman Coulter, CytoFLEX).

### Lymphocyte isolation and T cell-activation assay

Blood leukopaks were obtained from healthy people at the Shanghai Zhaxin Hospital of Integrated Traditional Chinese & Western Medicine under institutional review board-approved protocols. Peripheral blood mononuclear cells (PBMCs) were isolated according to the manufacturer’s instructions (Ficoll 400, F8636, Sigma–Aldrich). Human CD4 + and CD8 + T cells were purified using BD IMag anti-human CD4 (No. 557767) and anti-human CD8 beads (No. 557766), CD3-CD56 + NK cells were prepared by separation with magnetic beads (NK purification kit, Miltenyi Biotec), and the purities were confirmed with flow cytometry (Beckman Coulter, CytoFLEX). For a cell proliferation assay, CD8 + T cells were labeled with 10 μM carboxyfluorescein succinimidyl ester (CFSE, Invitrogen) according to the manufacturer’s protocol and assayed by flow cytometry. To determine the IFNγ secretion activity of the lymphocytes, 96-well cell culture plates (Corning) were pretreated with an anti-CD3 antibody (clone: OKT3, 0.4 μg/mL) for CD4 + T and CD8 + T cells and 100 U/mL recombinant human IL-2 (rhIL-2, PeproTech) for NK cells. After washing twice with PBS, 1 × 10^5^ CD4 + T, CD8 + T or NK cells in 200 μL of complete RPMI-1640 medium were added and treated with HuB6, urelumab or utomilumab across a range of doses (0.04 μg/mL, 0.4 μg/mL and 4 μg/mL). After 3 days of culture in a CO2 incubator at 37 °C, the IFN-γ content in the cell culture supernatant was determined with an ELISA kit according to the manufacturer’s manual (BioLegend).

### CD8 + T cell assay with FcγR crosslinking

To test 4-1BB agonist activity dependent on FcγR, CD8 + T cells were adjusted to 2 × 10^4^ cells/well and cocultured with CHO-K1 cells expressing different FcγRs at 1.0 × 10^4^ cells/well in a 96-well microplate bounded with 0.4 µg/mL anti-CD3 antibody (OKT3). Then, serially diluted antibodies were incubated with cocultured cells for 3 days in a CO2 incubator at 37 °C. After incubation, the secreted IFN-γ and IL-2 levels in the cell supernatants were measured by ELISA.

### Cytokine release analysis

For the cytokine release assay, PBMCs from 5 healthy donors (2 × 10^5^ cells/well) in RPMI-1640 medium with 10% FBS in 96-well flat-bottom plates were treated with 10 µg/mL of the tested antibodies. HuB6 was compared with an isotype control human IgG4 as well as positive control antibody (OKT3). After 48 h of incubation, the levels of the cytokines IFN-γ, TNF-α, IL-10, IL-2, IL-6, IL-4 and IL-17A in the culture medium were measured by cytometric bead array assay (C60021, QuantoBio) according to the manufacturer’s instructions. Fluorescence signals were measured by a CytoFlex system (Beckman).

### Model mouse

To establish two types of CRC tumor-grafted mouse models, 8-week-old human 4-1BB/4-1BBL double knock-in C57BL/6 and B-NDG B2m KO plus mice, in which human PBMCs were transplanted to reconstitute human immune cells, were purchased from Biocytogen Corporation (Beijing, China) and used according to the appropriate experimental protocol. Briefly, 2 × 10^6^ MC38 or CT26 cells mixed with Corning Matrigel in a 1:1 volume ratio were inoculated subcutaneously into human 4-1BB/4-1BBL double knock-in C57BL/6 mice. Similarly, 2 × 10^6^ Colo205 cells mixed with 1:1 Matrigel were inoculated subcutaneously into B-NDG B2m KO plus mice. After palpable tumors were established, the mice were randomized on the basis of tumor volume and body weight. Subsequently, treatment with a mAb or an isotype control was performed twice a week for up to 3 weeks by intraperitoneal injection. Tumor growth was monitored twice a week by measuring tumor length and width. Tumor volume was calculated according to the following equation: 0.5 × length × width × width.

### Toxicology study

HuB6 toxicity studies were conducted with humanized model mice and purpose-bred cynomolgus monkeys. Humanized 4-1BB mice were intraperitoneally injected with a low dose (3 mg/kg) or a high dose (30 mg/kg) of HuB6, utomilumab, urelumab or an isotype antibody once every 3 days for 6 total injections. Cynomolgus monkeys were administered repeated intravenous doses of 3, 10, and 30 mg/kg/week for 5 weeks or single doses of 60 and 180 mg/kg for toxicity studies. Two male and 2 female cynomolgus monkeys were randomly assigned to each group, and the antibodies were administered via intravenous infusion at a dose of 5 mL/kg administered at a rate of 1 mL/min. Mouse necropsies were performed according to a standard protocol, and the major organs were collected for histological evaluation. All the tissues were fixed in 10% neutral-buffered formalin, routinely processed, embedded in paraffin, sectioned, stained with hematoxylin and eosin (HE) and analyzed by a professional pathologist. In addition, blood and serum were collected for clinical hematological and chemical analyses using a Sysmex BX4000.

### Statistical analysis

Statistical analyses were performed using GraphPad Prism 8.0 (GraphPad Software), and one-way or two-way ANOVA was used to compare intergroup differences. A p value of < 0.05 was considered significant.

## Results

### Characterization of the mAb HuB6

Previous reports have demonstrated that the IgG4 form of recombinant human IgG mAbs is useful in various therapeutic applications to reduce FcγR activation and Fc-mediated toxicity, including the complement-dependent cytotoxicity (CDC) and antibody-dependent cell-mediated cytotoxicity (ADCC) pathways [12, 13]. Therefore, we first screened twelve humanized 4-1BB-targeted single-chain variable fragments (scFvs) belonging to the IgG4 subtype derived from a hybridoma mAb (No. 37G10F4, patents CN112794904A and CN112794906A) with high affinity for the human 4-1BB ECD and physiological activity in activating T cell functions using artificial intelligence computer aided design technology, and then the superior candidate mAb HuB6 was generated from the scFvB60103 colony using unbiased functional screening.

**Fig. 1:**
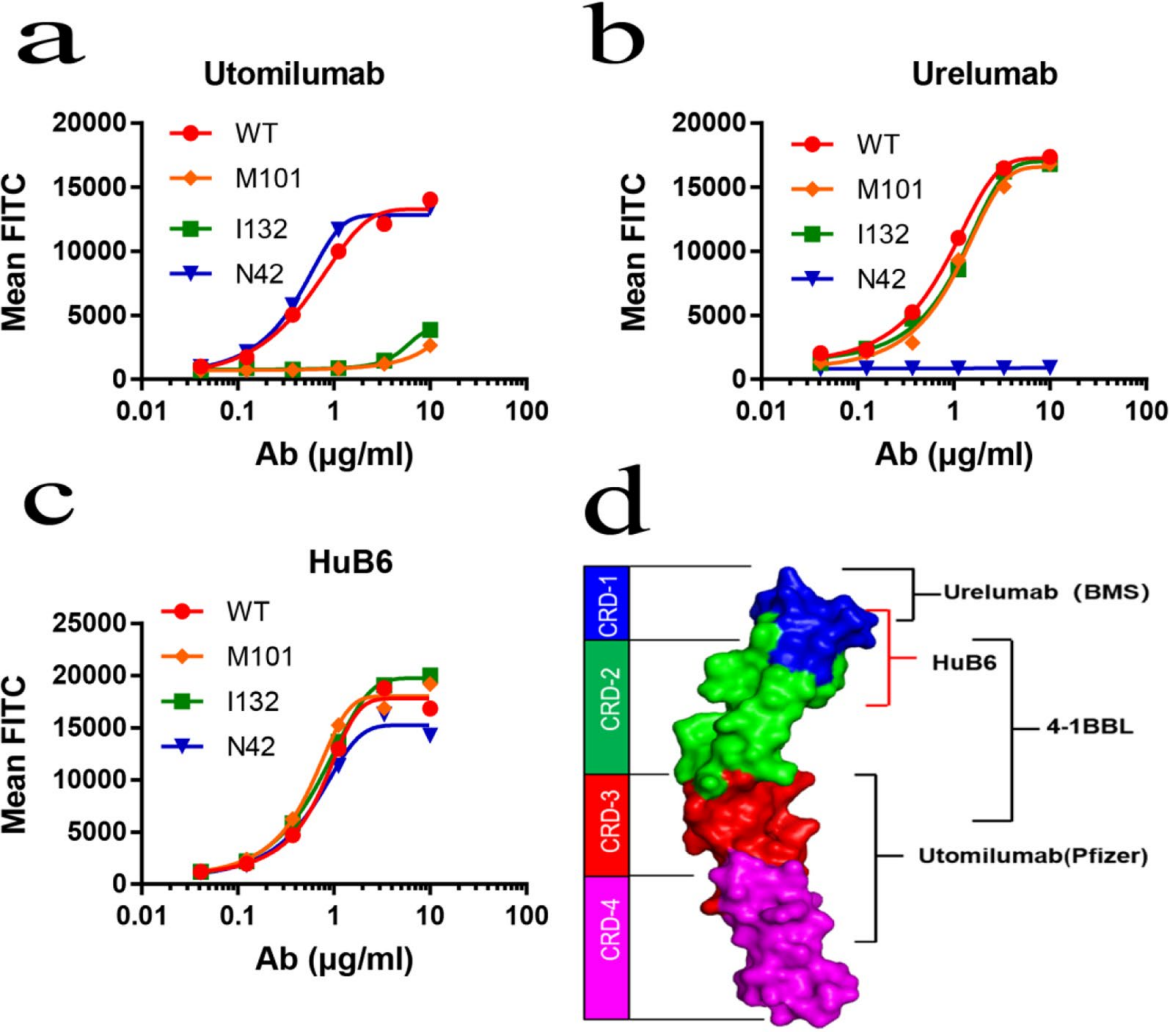
HuB6 binds a unique epitope within 4-1BB. The key binding sites of **a** utomilumab, **b** urelumab and **c** HuB6 were analyzed with an amino acid point mutation binding assay using flow cytometry. The results are representative of three different experiments and expressed as the mean values. Hu4-1BB antigens with a mutation in the key amino acid M101, I132 or N42 were transfected into Expi293 cells. **d** The binding epitopes of utomilumab, urelumab and HuB6 are shown on the structural model of 4-1BB

To determine the possible binding epitope of the mAb HuB6, important extracellular amino acid sites in the human 4-1BB antigen were mutated, and the affinity changes were analyzed. It is known that amino acid I64 of the 4-1BB protein is the key binding site for 4-1BBL, M101 and I132 are the binding amino acids for utomilumab, and N42 is fundamental for urelumab binding [10]. Therefore, the mutations in the 4-1BB antigen were targeted to the key amino acids M101, I132 and N42 by making point mutations, and the mAb-bound epitopes were analyzed using flow cytometry. Our results showed that utomilumab hardly bound the M101-mutated or I132-mutated 4-1BB antigen (Fig. 1a) and urelumab did not bind the N42-mutated antigen (Fig. 1b), but HuB6 could bind all the mutated antigens (Fig. 1c). Therefore, the epitope recognized by HuB6 exists in both cysteine-rich domain 1 (CRD1) and CRD2 of human 4-1BB, which is unique and distinct from those recognized by utomilumab (CRD3 and CRD4) and urelumab (CRD1) (Fig. 1d, Additional file 1: Fig. S1).

**Fig. 2:**
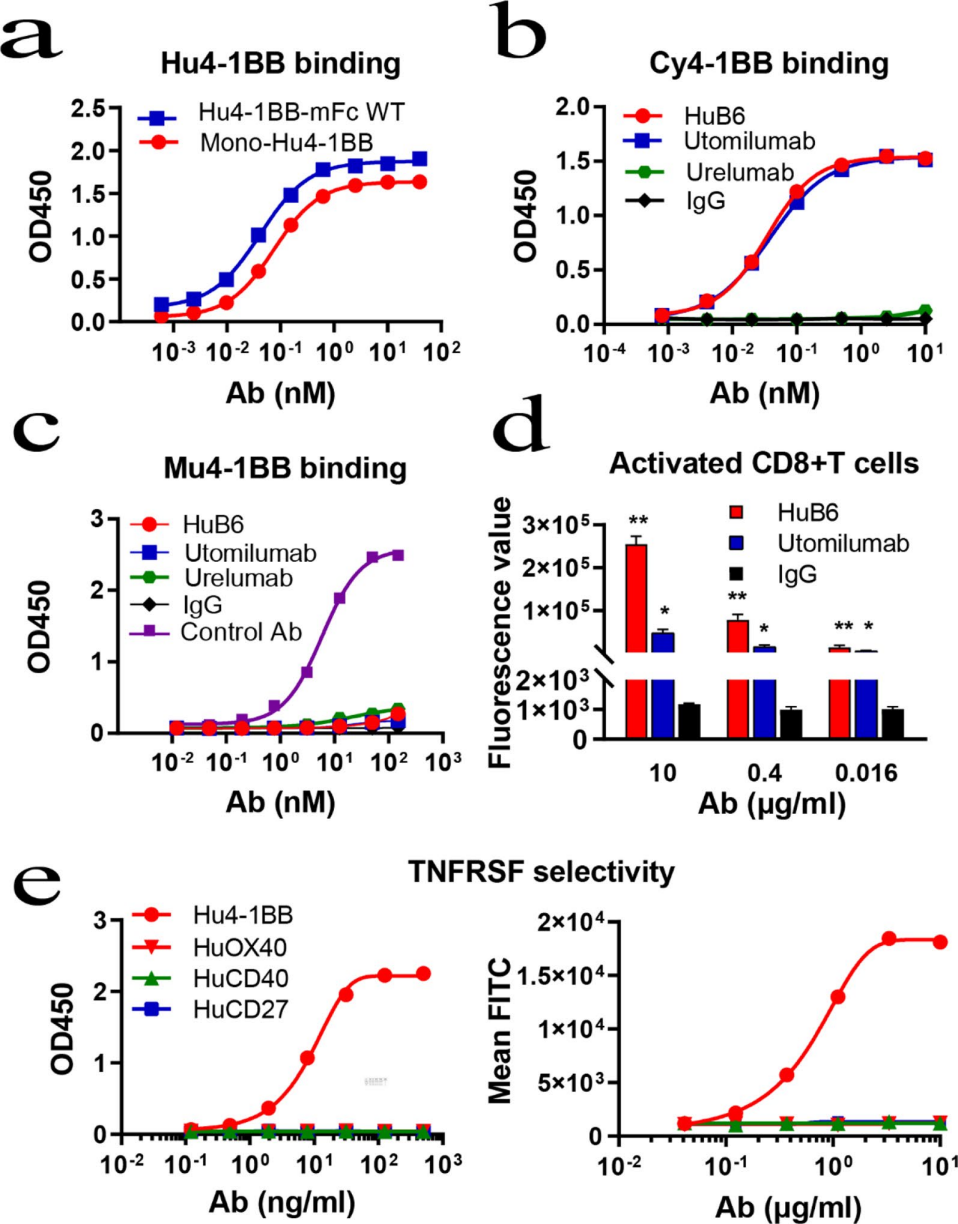
HuB6 has high binding specificity and affinity. **a** HuB6 bound to both the monomeric form (mono-Hu4-1BB) and dimeric form (Hu4-1BB-mFc WT) of human 4-1BB, as determined by ELISA. **b** The dynamic curves for HuB6, utomilumab and urelumab binding to cynomolgus monkey 4-1BB (Cy4-1BB) determined by ELISA. **c** The dynamic curves for HuB6, utomilumab and urelumab binding to murine 4-1BB (Mu4-1BB) determined by ELISA. **d** The fluorescence values of activated CD8 + T cells binding with FITC-labeled HuB6, utomilumab or an isotype antibody determined by flow cytometry. The results are representative of three different experiments and expressed as the mean value ± SD. **p < 0.001 compared to the utomilumab group. *p < 0.001 compared to the IgG group. **e** HuB6 binding selectivity to TNFRSF members including 4-1BB, OX40, CD40 and CD27 was evaluated using flow cytometry and ELISA

To define the specificity and affinity of HuB6, both the monomeric form (mono-Hu4-1BB) and dimeric form (Hu4-1BB-mFc WT) of the human 4-1BB ECD were prepared, and HuB6 showed different binding affinities [half-maximal effective dose (EC50): 0.086 nM for mono-Hu4-1BB, 0.046 nM for Hu4-1BB-mFc WT; Fig. 2a]. Furthermore, HuB6 could bind to the 4-1BB protein of cynomolgus monkeys with an affinity similar to that of utomilumab (HuB6 EC50: 0.035 nM, utomilumab EC50: 0.038 nM), but urelumab did not show any affinity (Fig. 2b). Moreover, none of the three 4-1BB-specific antibodies cross-reacted with mouse 4-1BB, while the control anti-mouse 4-1BB mAb could bind to mouse 4-1BB (Fig. 2c). By a flow cytometry assay, HuB6 was found to bind to activated CD8 + T cells with higher affinity than utomilumab at concentrations ranging from 0.016 μg/mL to 10 μg/mL (Fig. 2d). The Kd value of HuB6 binding to human 4-1BB was similar to that of utomilumab but higher than that of urelumab, as determined by the Biacore and ForteBio methods. Furthermore, through competitive binding experiments, 4-1BBL was shown to compete with HuB6 and utomilumab for binding to 4-1BB in a concentration-dependent manner, but no competition was observed for urelumab (Table 1). These results indicate that HuB6 has high antigen specificity and affinity, similar to utomilumab. In addition, HuB6 was confirmed to bind selectively to 4-1BB, not other human TNFRSF members (OX40, CD40 and CD27) using flow cytometry and ELISA (Fig. 2e).

**Table 1:** Comparison of the binding affinities (Kd, n = 3) of mAbs to human 4-1BB

Antibody	Kon (1/Ms)	Koff (1/s)	Kd (nM, SPR)	Kd (nM, BLI)	4-1BBL competition
Urelumab	209,700	0.0011	5.34 ± 0.49	5.76 ± 0.85	No
Utomilumab	504,900	0.0089	17.69 ± 1.92	22.12 ± 3.75	Yes
HuB6	94,260	0.0018	18.56 ± 2.49	13.98 ± 2.60	Yes

Yes/No indicates whether 4-1BBL can compete for binding to 4-1BB

### HuB6 increases T cell activation dependent on FcR crosslinking

**Fig. 3:**
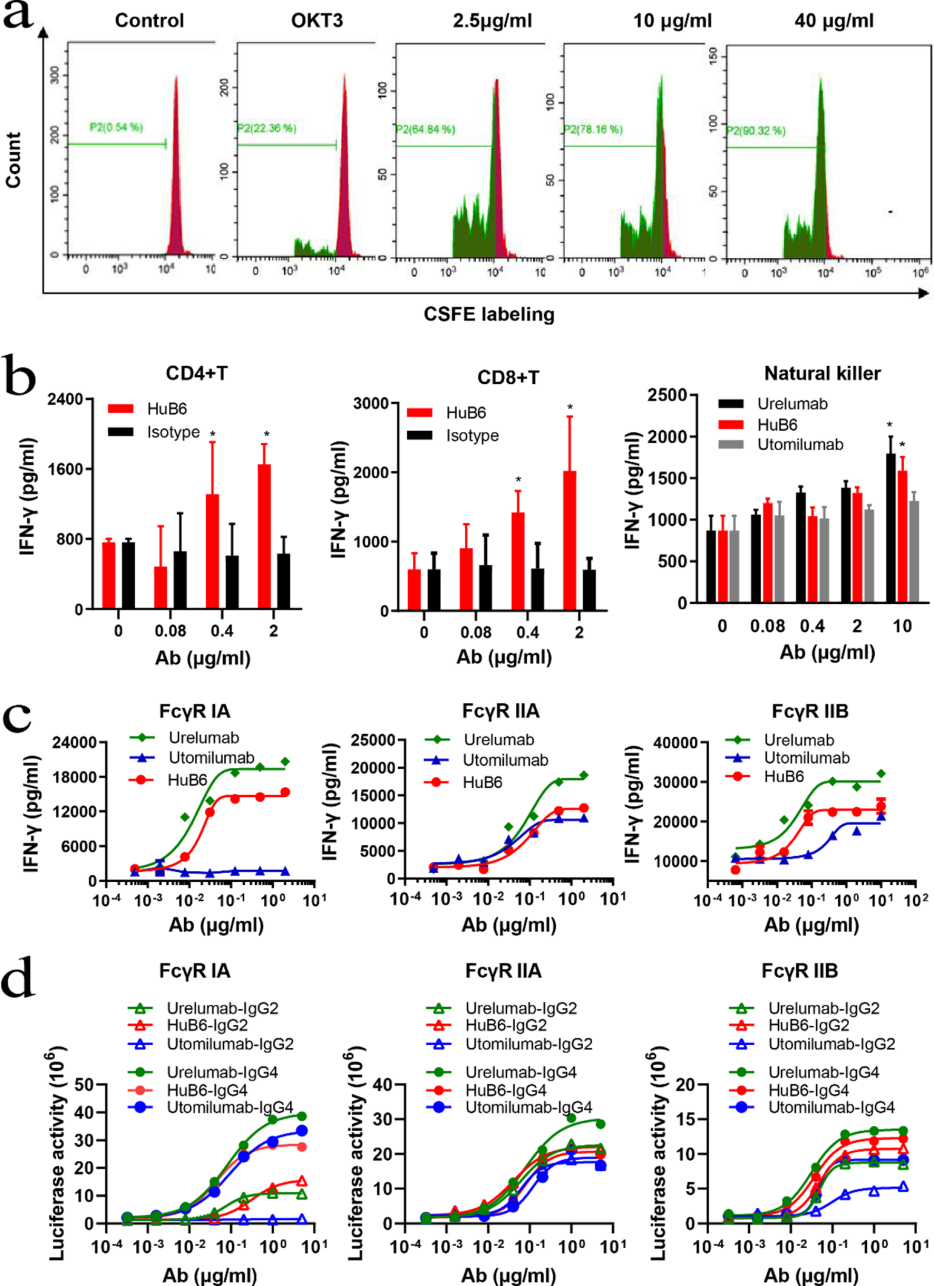
HuB6 promotes the proliferation and activation of T cells in a manner dependent on FcγR crosslinking. **a** The proliferation of CD8 + T cells was induced by HuB6, and the cell percentage was determined by CSFE labeling. HuB6 was used at three concentrations: 2.5, 10 and 40 μg/ml. **b** CD4 + T, CD8 + T and natural killer (NK) cell activation by HuB6 at the indicated concentrations was monitored through detection of INF-γ in the supernatant by ELISA. *p < 0.01 compared to the utomilumab group. **c** The activation of CD8 + T cells by HuB6, urelumab or utomilumab dependent on FcγR crosslinking in the presence of FcγRIA, FcγRIIA or FcγRIIB was monitored through detection of INF-γ in the supernatant by ELISA. **d** The activities of HuB6, urelumab and utomilumab engrafting the Fab into Fc with different subclasses (IgG2, IgG4) were compared using the functional cellular NFκB reporter assay. The results are representative of three different experiments and expressed as the mean values

It was reported that antibodies targeting 4-1BB can enhance the proliferation of antigen-stimulated T cells in vitro and promote CD8 + T cell-dependent antitumor immunity in preclinical cancer models [14]. Here, the effects of the mAb HuB6 on CD8 + T cells were tested. By the CFSE labeling method, the percentages of proliferating cells were shown to be 64.84%, 78.16% and 90.32% after treatment with HuB6 at 2.5 μg/mL, 10 μg/mL and 40 μg/mL, respectively; in comparison, the percentages in the blank control and only anti-CD3 antibody treatment groups were 0.54% and 22.36%, respectively (Fig. 3a). Moreover, HuB6 increased the secretion of IFN-γ by CD8 + T, CD4 + T and NK cells in a dose-dependent manner (Fig. 3b).

A previous study indicated that the FcγR interaction is critical for the bioactivity of 4-1BB-specific mAbs [15]. First, the affinities of HuB6 to different types of human FcγRs were analyzed, only FcγRIA had a positive binding (Kd = 8.86 ± 2.20 nM) and FcγRIIB or FcγRIIA had no measurable binding within the tested concentrations (Table 2). Then, the effect of Fc-mediated crosslinking on HuB6 was evaluated using different FcγRs expressed on CHO-K1 cells. HuB6 activated CD8 + T cells to produce IFNγ in the presence of FcγRIA, FcγRIIB or FcγRIIA, similar to the IgG4 isotype mAb urelumab, although the activation extent was lower than that achieved with urelumab. In comparison, the IgG2 mAb utomilumab exhibited an activating function in the presence of FcγRIIB or FcγRIIA but little activity in the presence of only FcγRIA (Fig. 3c). It has been reported that IgG of all 4 subclasses has different affinities for FcγRs, and the higher efficacy of IgG4 over IgG2 is likely due to its increased binding affinity to FcγRs [16, 17]. Therefore, the activities of HuB6, urelumab and utomilumab engrafting the Fab into Fc with different subclasses (IgG2, IgG4) were compared using the functional cellular NFκB reporter assay. As expected, all IgG4 mAbs had stronger 4-1BB agonist activity than their IgG2 counterparts, and urelumab-IgG4 had the strongest activity. Notably, the activity of HuB6 was equivalent to that of urelumab regardless of the subclass for IgG4 or IgG2 in the presence of FcγRIA, while utomilumab-IgG2 did not even activate 4-1BB signaling. Moreover, the activity of HuB6 remained stable and showed few changes after it was converted from IgG4 to IgG2 in the presence of FcγRIIB or FcγRIIA (Fig. 3d). In addition, in the absence of any FcγR, no activity was observed for HuB6 or utomilumab, whereas urelumab could still activate CD8 + T cells (Additional file 1: Fig. S2). These data indicated that HuB6 should induce T cell activation in a manner dependent on FcγR crosslinking and FcγRIA may be the major driver for the activity and safety of HuB6, which is distinct from urelumab and utomilumab.

**Table 2:** SPR binding affinities (Kd, n = 3) of HuB6 to human FcγRs

FcγR type	K_on_ (1/Ms)	K_off_ (1/s)	K_d_ (nM)
IA	531,000	0.0045	8.86 ± 2.20
IIA	NB	NB	NB
IIB	NB	NB	NB
IIIA (F176)	NB	NB	NB
IIIA (V176)	NB	NB	NB

NB, no measurable binding within the tested concentrations

### HuB6 exerts potent antitumor effects

**Fig. 4:**
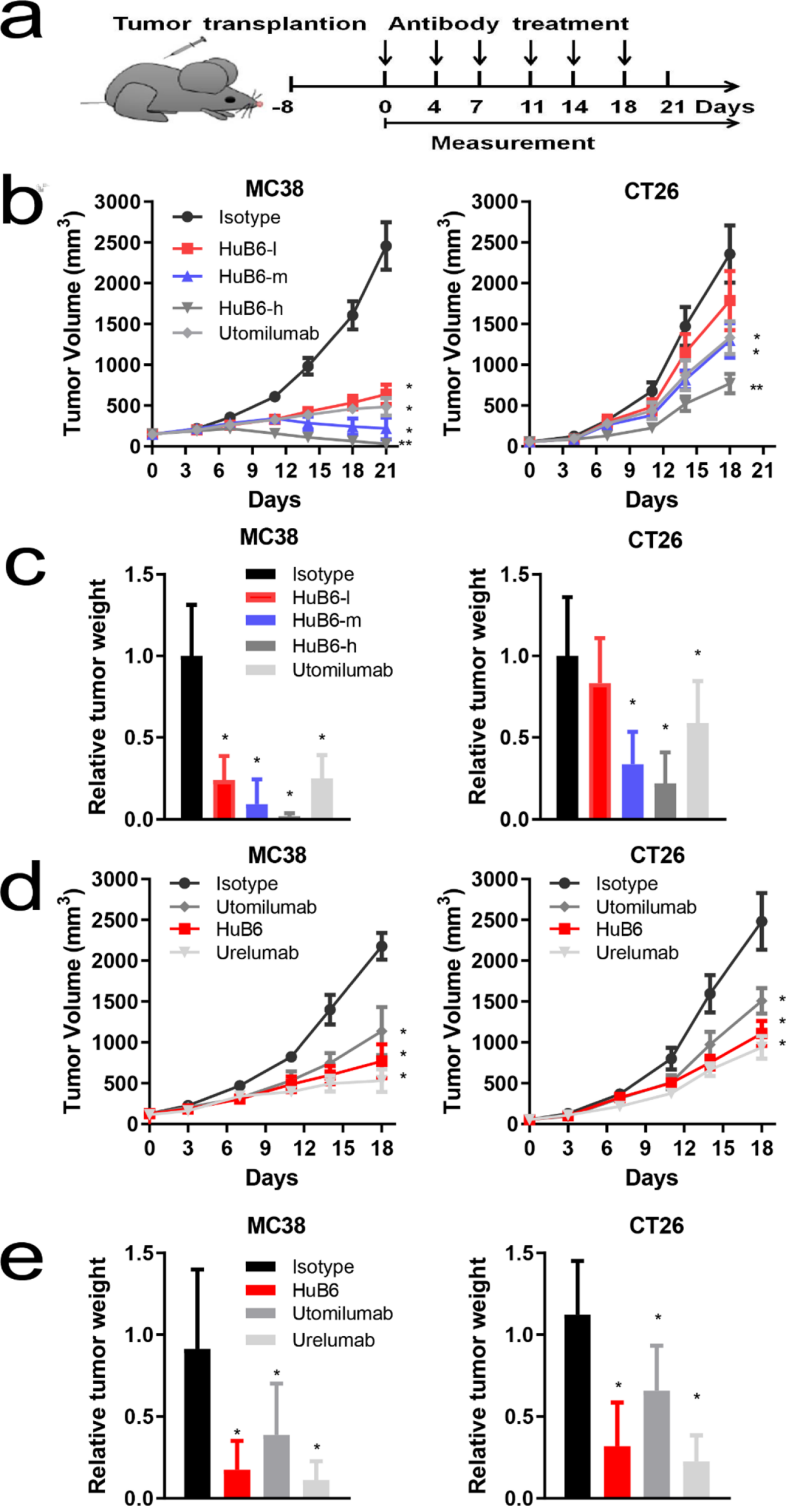
HuB6 exerts potent antitumor activities in humanized mouse models. **a** Schematic diagram for in vivo antibody treatment of human 4-1BB knock-in mice bearing murine colorectal cancer transplants or human PBMC-engrafted mice bearing human colorectal cancer transplants. After tumors were established, the mice were randomized into groups of 8 animals per group on day 8. Treatment with HuB6 orutomilumab was administered six times (indicated by vertical arrows) to MC38 model mice or five times to CT26 model mice. **b** The changes in tumor volume in the HuB6 (MC38 model: l, 0.3 mg/kg; m, 1 mg/kg; and h, 3 mg/kg. CT26 model: l, 1 mg/kg; m, 3 mg/kg; and h, 10 mg/kg) and utomilumab (MC38 model: 1 mg/kg; CT26 model: 10 mg/kg) groups. **c** Relative tumor weight determined by comparison with the isotype control. Tumor tissues were removed and weighed at the end of the experiment in (**b**). **d** Comparison of tumor volume after the treatment with HuB6, utomilumab and urelumab (MC38 model: 0.3 mg/kg; CT26 model: 1 mg/kg). **e** Relative tumor weight determined by comparison with the isotype control. Tumor tissues were removed and weighed at the end of the experiment in (**d**). In all panels, n = 8 biologically independent animals. Statistical analysis was performed using two-way ANOVA; *p < 0.05 compared to the isotype group, **p < 0.05 compared to the single antibody group. Error bars within the figure represent the mean ± SD

Humanized mouse models bearing CRC tumors were established to evaluate the antitumor effect of HuB6 in vivo*,* and a schematic diagram of HuB6 or control mAb treatment is shown in Fig. 4a. First, a dose escalation study was performed with HuB6 in humanized 4-1BB mouse models bearing MC38 or CT26 tumors, and the HuB6-treated groups showed dose-dependent antitumor effects on both tumor volume (p < 0.05, Fig. 4b) and tumor weight (p < 0.05, Fig. 4c). Moreover, the average tumor volumes in the high-dose HuB6 group were significantly smaller than those in the middle-dose HuB6 or utomilumab group (p < 0.05) for both MC38 and CT26 models. Notably, some tumors in the MC38 model completely regressed in all three HuB6 groups, showing a dose-dependent relationship (1, 2 and 3 tumors receded in the low-, middle- and high-dose groups, respectively) at the end of 21-day observation, whereas no complete tumor regression was observed in the utomilumab group (Additional file 1: Fig. S3a). Second, the low-dose HuB6 was chosen in comparison with the same dosage of urelumab and utomilumab and the in vivo results demonstrated that HuB6 had a similar antitumor effect with the two control mAbs (p > 0.05) and that either tumor volume or tumor weight in HuB6 group was significantly reduced compared with that of isotype group for both MC38 and CT26 models (p < 0.05, Fig. 4c, d and Additional file 1: Fig. S3b).

### HuB6 induces antitumor immune memory and enhances efficacy when combined with anti-PD-L1 mAb

**Fig. 5:**
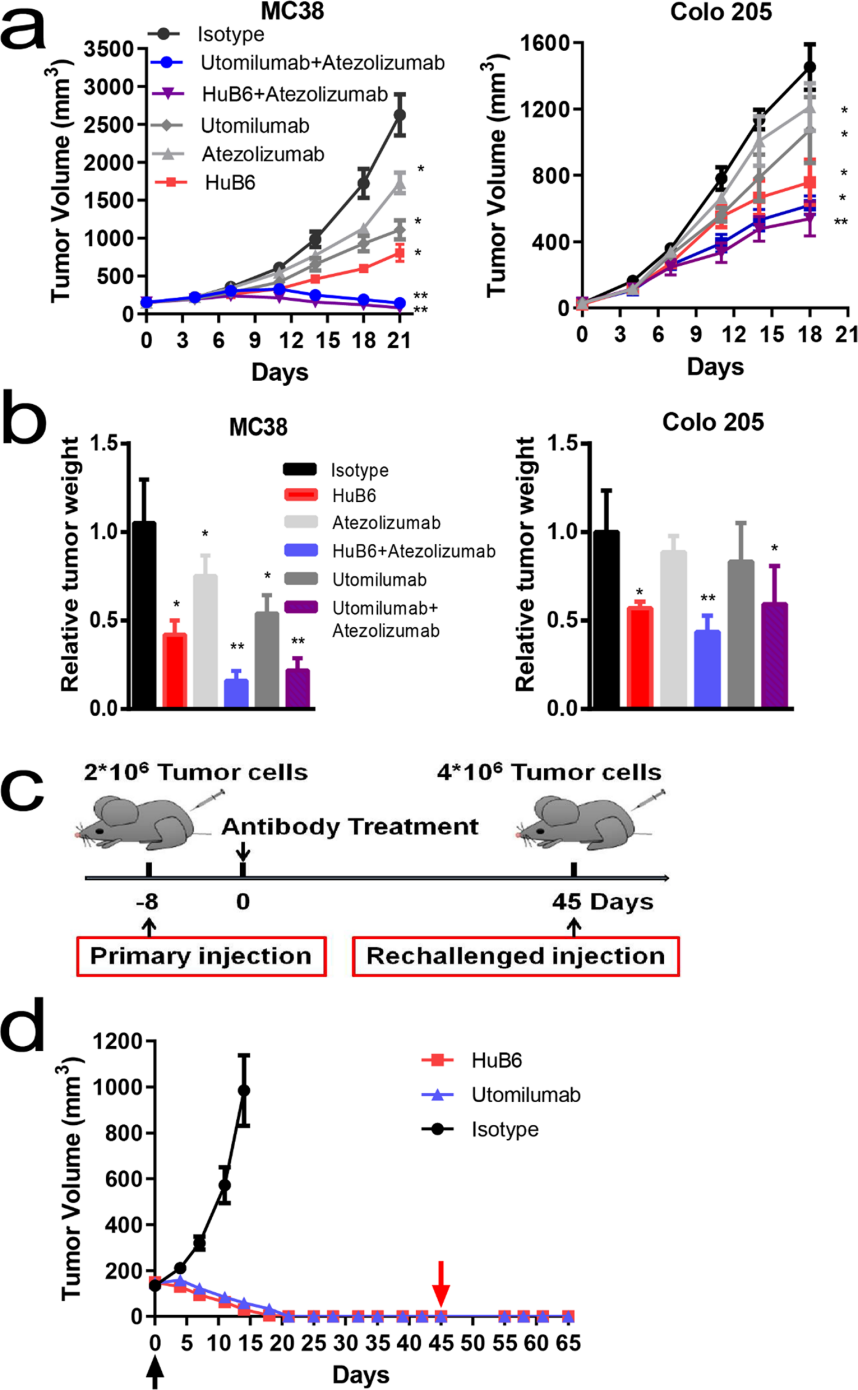
HuB6 induces antitumor immune memory and enhanced efficacy when combined with anti-PD-L1 mAb. **a** Changes of tumor volume after the treatment with HuB6 (MC38 model: 0.3 mg/kg, n = 5; Colo205 model: 10 mg/kg, n = 8) combined with atezolizumab (MC38 model: 0.5 mg/kg; Colo205 model: 10 mg/kg). **b** Relative tumor weight determined by comparison with the isotype control. Tumor tissues were removed and weighed at the end of the experiment in (**a**). **c** Schematic of the experimental design for testing antitumor immune memory. **d** Tumor volume changes in humanized 4-1BB mice subcutaneously inoculated with 2 × 10^6^ MC38 cells and then intraperitoneally injected with 10 mg/kg HuB6 or utomilumab only once eight days later; the mice were given a secondary subcutaneous injection of 4 × 10^6^ MC38 cells on day 45 (n = 6). Statistical analysis was performed using two-way ANOVA; *p < 0.05 compared to the isotype group, **p < 0.05 compared to the single antibody group. Error bars within the figure represent the mean ± SD

Next, the combined antitumor effect of HuB6 and an anti-PD-L1 mAb was investigated. It was reported that the MC38 cell line has a positive response to the anti-PD-L1 mAb atezolizumab [18]. Therefore, the humanized 4-1BB mouse model bearing MC38 tumors was chosen and treated with 1 mg/kg atezolizumab alone, 0.3 mg/kg HuB6 or utomilumab alone and their combinations. As shown in Fig. 5a, although atezolizumab alone inhibited tumor growth (p < 0.05), combination with HuB6 or utomilumab significantly improved the antitumor effect (p < 0.05), and one tumor completely regressed in the HuB6 plus atezolizumab group. Similarly, HuB6 plus atezolizumab produced an enhanced antitumor effect in human PBMC-engrafted mice bearing human CRC cell line Colo205 transplants compared with either mAb monotherapy (p < 0.05). Furthermore, by measuring tumor weight, a remarkable difference was found between atezolizumab combined with HuB6 or utomilumab and any mAb alone in the MC38 model (p < 0.05), but a significant difference was only found between atezolizumab plus HuB6 and atezolizumab alone in the Colo205 model (p < 0.05, Fig. 5b).

According to the reported experimental procedure for evaluating antitumor immune memory [19], we tested the ability of HuB6 to induce immune memory against cancer (Fig. 5c). First, humanized 4-1BB mice were inoculated with 2 × 10^6^ MC38 cells in one flank and then intraperitoneally injected with 10 mg/kg HuB6, utomilumab or an isotype control only once when the tumor volume reached approximately 150 mm^3^ (eight days later). Remarkably, all the treated mice exhibited complete tumor regression on the 18^th^ day in the HuB6 group or on the 21^st^ day in the utomilumab group. Second, on the 45^th^ day, the surviving tumor-free mice were rechallenged with 4 × 10^6^ MC38 cells injected into the other flank and showed complete tumor rejection (Fig. 5d).

### HuB6 does not induce the production of proinflammatory cytokines

**Fig. 6:**
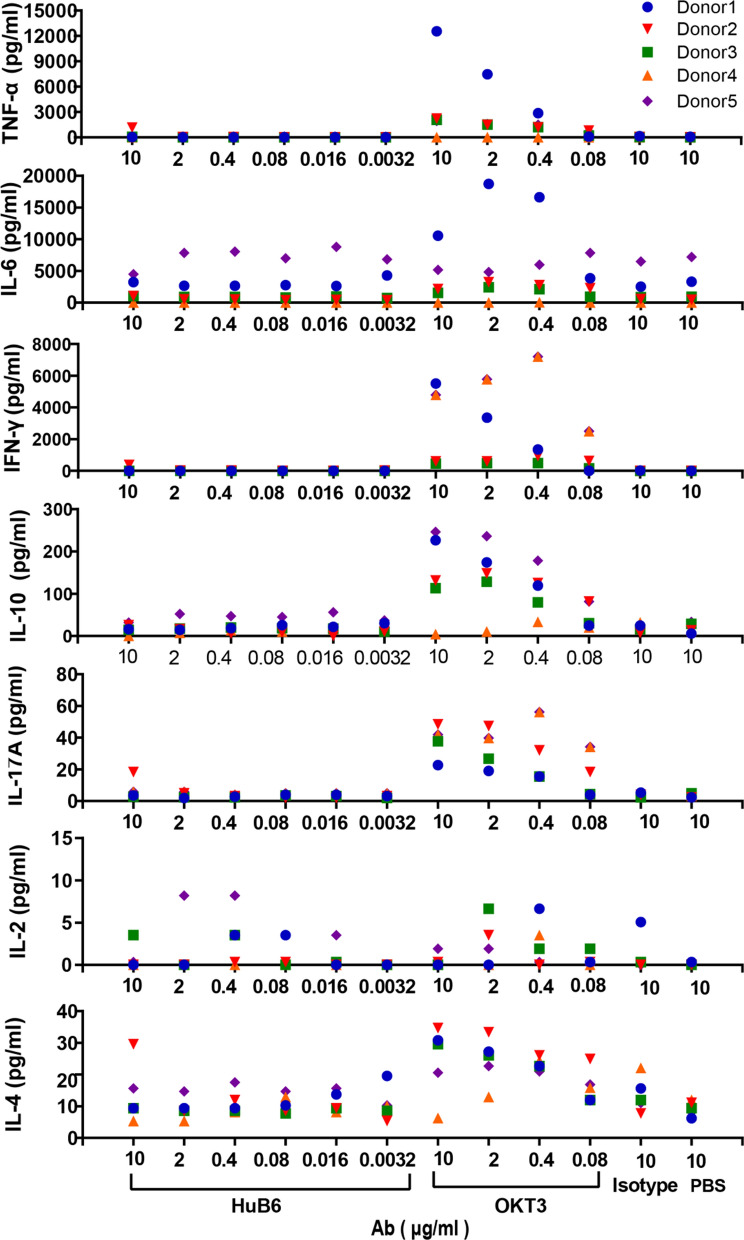
HuB6 does not induce the production of proinflammatory cytokines. The levels of the cytokines IFN-γ, TNF-α, IL-10, IL-2, IL-6, IL-4, and IL-17A in the culture medium with PBMCs (2 × 10^5^ cells/well) from 5 healthy donors treated with 10 µg/mL of the tested antibodies for 48 h were measured by cytometric bead array assay. HuB6 was compared with the isotype control human IgG4 and the positive control antibody OKT3

To further dissect the possible toxicity of HuB6 in vitro, nonspecific production of various inflammatory cytokines, including TNF-ɑ, IFN-γ, IL-2, IL-4, IL-6, IL-10 and IL-17A, was examined in the PBMCs of 5 healthy donors treated with HuB6 and the control. The results showed that the average levels of cytokines induced by HuB6 were similar to those of the isotype and PBS groups and significantly lower than those of the positive control OKT3 group, which confirmed that HuB6 did not induce nonspecific proinflammatory cytokines in the absence of T cell receptor (TCR) stimulation (Fig. 6).

### HuB6 exhibits high safety in animal models

**Fig. 7:**
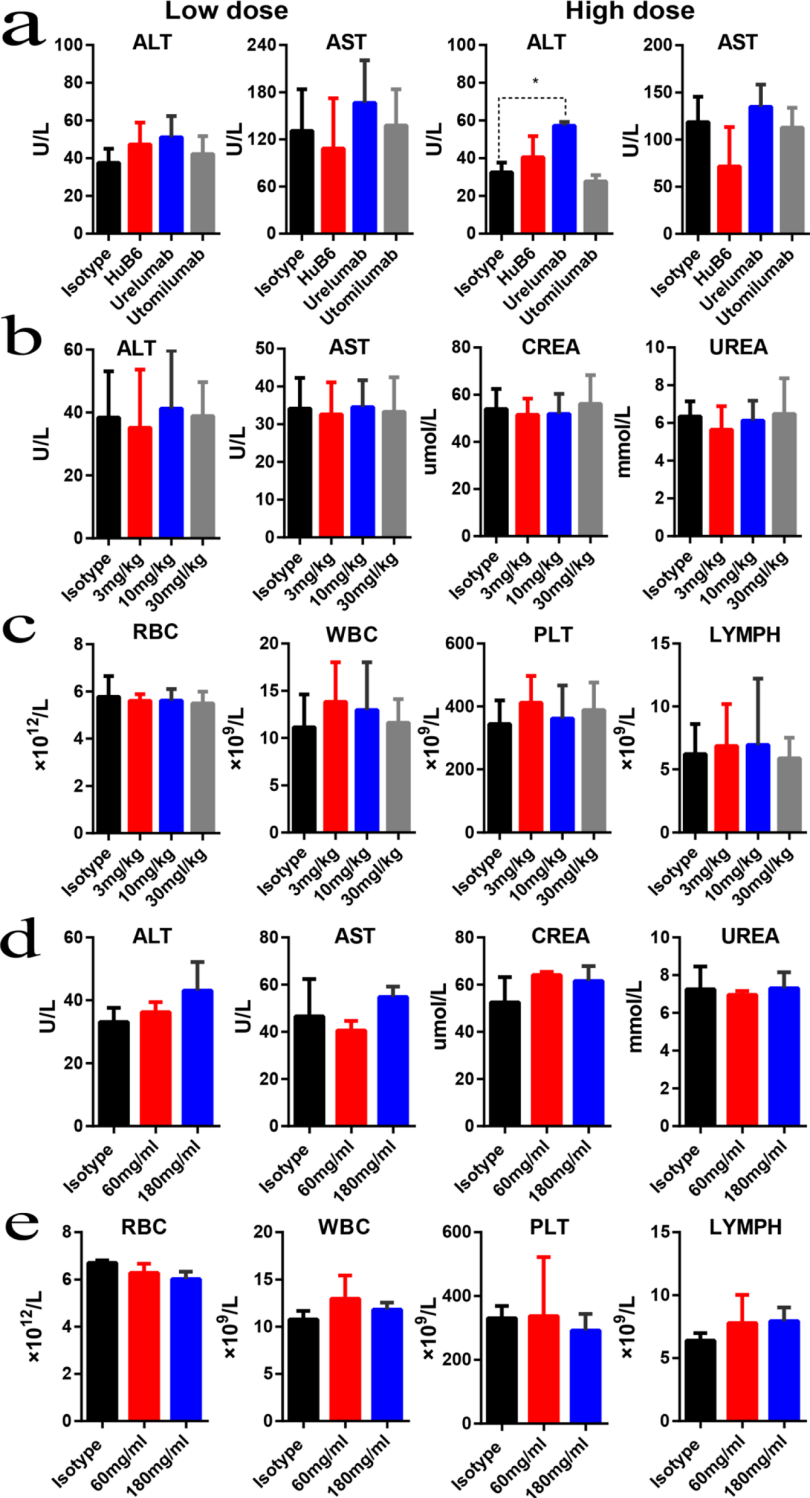
HuB6 has a good safety profile. **a** Humanized 4-1BB model mice were intraperitoneally injected with HuB6, utomilumab, urelumab or an isotype control once every 3 days (6 times total). The serum levels of alanine aminotransferase (ALT) and aspartate aminotransferase (AST) in the low-dose (3 mg/kg) group and high-dose (30 mg/kg) group were measured on the 16th day. *p < 0.001. **b**, **c** Eight male and 8 female cynomolgus monkeys were randomly divided into the low-dose (3 mg/kg), middle-dose (10 mg/kg) and high-dose (30 mg/kg) HuB6 groups and an isotype group, and the antibodies were administered via repeated intravenous infusions (once a week for 5 weeks). The evaluated serum biochemical markers included ALT, AST, creatinine (CREA) and UREA (**b**), and the hematological indexes included red blood cells (RBCs), white blood cells (WBCs), platelets (PLTs) and lymphocytes (LYMPHs) (**c**). **d**, **e** Six male and 6 female cynomolgus monkeys were randomly divided into the low-dose (60 mg/kg) and high-dose (180 mg/kg) HuB6 groups and an isotype control group, and the antibodies were administered only once via intravenous infusion. The evaluated serum biochemical markers included ALT, AST, CREA and UREA (**d**), and the hematological indexes included RBCs, WBCs, PLTs and LYMPHs (**e**)

To assay the possible toxicity of HuB6 in vivo, humanized 4-1BB mice and cynomolgus monkeys were employed. Humanized 4-1BB mice were intraperitoneally injected with a low dose (3 mg/kg) or a high dose (30 mg/kg) of HuB6, utomilumab, urelumab or an isotype control once every 3 days (6 times total), and no significant differences in hematological markers, including alanine aminotransferase (ALT) and aspartate aminotransferase (AST), were found between HuB6 or utomilumab and the isotype control in either the low-dose or high-dose group (p > 0.05, Fig. 7a). However, the ALT level in the high-dose urelumab group was significantly higher than that in the isotype group on the 18^th^ day at the end of the experiment (p < 0.001, Fig. 7a). In addition, histopathological evaluation of major organs, including the heart, liver, lung, kidney and spleen, confirmed the high safety of HuB6 (Additional file 1: Fig. S4).

For the cynomolgus monkey study, 5-week repeat-dose toxicity and single-dose toxicity tests were performed. First, 8 males and 8 females were randomly divided into HuB6 groups treated with a low dose (3 mg/kg), middle dose (10 mg/kg) or high dose (30 mg/kg) and an isotype control group, and the antibodies were administered via repeated intravenous infusions (once a week for 5 weeks) at a dose of 5 mL/kg administered at a rate of 1 mL/min. Hematological indexes, including red blood cells (RBCs), white blood cells (WBCs), platelets (PLTs) and lymphocytes (LYMPHs), and serum biochemical markers, including ALT, AST, creatinine (CREA) and urea, were determined, and no abnormal changes were observed in any of the groups on day 35 (Fig. 7b, c). In our study, HuB6 was well tolerated up to a dosage of 30 mg/kg/week without any abnormalities in general condition (e.g., decreased food consumption or body weight) or hematological changes (e.g., decrease in neutrophils or PLTs). In contrast, utomilumab, despite having much weaker agonistic activity, was reported to cause dose-limiting toxicity at dosages greater than 5 mg/kg/week [11] and elicited systemic toxicity at a dosage of 30 mg/kg/week in a 4-week toxicity study [8]. Moreover, we conducted a single-dose toxicity study with cynomolgus monkeys. HuB6 was well tolerated at 60 mg/kg without any abnormalities, and when the dose reached 180 mg/kg, mild and transient liver toxicity (less than twofold increases in ALT and AST) was found on day 2. The indexes were restored to normal on day 9, and no other abnormalities occurred (Fig. 7d, e). These results demonstrate that HuB6 has a good safety profile.

## Discussion

In this study, we demonstrated that the humanized anti-4-1BB mAb Hub6 induced T cell proliferation and activity and had potent efficacy in tumor inhibition and immune memory induction without systemic toxicity; thus, it was regarded as a potential candidate for cancer immunotherapy. As 4-1BB is one of the costimulatory receptors of immune cells, 4-1BB-targeting mAbs have shown promising antitumor effects in preclinical models [20]. However, the clinical development of two leading molecules, utomilumab and urelumab, is facing serious challenges due to low efficacy or severe systemic toxicity [8, 11].

Recently, several bispecific tumor antigen-targeted 4-1BB agonists have been developed [21–24]. However, their therapeutic efficacy relies fully on the expression of tumor antigens, limiting their clinical application to patients with antigen overexpression. In addition, tumor antigen, such as EGFR, is widely expressed in normal, non-neoplastic tissues, and its use as a target antigen can lead to severe on-target, off-tumor immunotoxicity [21, 24]. It follows that the selected tumor antigens should be highly tumor-specific and their high expression is often limited to only specific types of cancer [8]. In contrast, the success of immune checkpoint inhibitors, such as anti–PD-1/PD-L1 antibodies, is partially attributed to their broad applicability in a variety of cancers regardless of the antigen expression status. Therefore, a novel humanized anti-4-1BB agonistic antibody that has strong agonistic activity, a high safety profile and broad applicability without depending on tumor antigen expression is urgently needed.

Our previous studies showed that HER2-targeted antibodies with different binding epitopes exhibited different antitumor properties [25, 26]. Here, we screened twelve humanized 4-1BB-targeted IgG4 subtype scFvs and then generated a novel anti-4-1BB mAb, HuB6, with an antigen epitope distinct from those of other known antibodies, such as utomilumab and urelumab; thus, HuB6 has unique antitumor efficacy and a high safety profile. As shown in Fig. 1d, the binding epitope of HuB6 is between CRD1 and CRD2 of the 4-1BB protein, while urelumab binds to CRD1, and utomilumab binds between CRD3 and CRD4. The binding site of 4-1BBL overlaps with those of HuB6 and utomilumab but not with that of urelumab; thus, both HuB6 and utomilumab are ligand-blocking antibodies, while urelumab is a non-ligand-blocking antibody, which was confirmed by the 4-1BBL competitive binding result and thus could allow HuB6 to activate T cells mimicking 4-1BBL. As a 4-1BB agonist, HuB6 increased the proliferation of CD8 + T cells and the production of the antitumor cytokine IFN-γ, inhibited tumor growth in all the mouse models tested, induced potent antitumor immune memory and exerted an enhanced tumor-inhibiting effect in combination with an anti-PDL1 mAb, similar to utomilumab, urelumab and several other potential therapeutics [8, 27–30]*.*

However, obvious differences in agonistic activity and toxicity are also easily found among these antibodies. First, the affinity of HuB6 for human 4-1BB was similar to that of utomilumab, but the tumor inhibitory efficacy in several mouse models seemed to be greater than that of utomilumab although the difference was not significant. Second, Hub6 was constructed as a recombinant human IgG4 mAb, which had more agonistic activity than its IgG2 counterpart (Fig. 3d). Moreover, Hub6 showed high safety in vitro and in vivo (Figs. 6, 7). However, as the same IgG4 mAb, urelumab caused severe liver toxicity, consistent with a previous study [9]. The above differences could be interpreted with epitope accessibility theory [10]. Namely, the binding site on the very N-terminus of CRD1 targeted by urelumab orients the antibody Fc domain to be optimally exposed for interaction with FcγR, which potentially enhances ADCC and CDC. The utomilumab epitope, which is closer to the cell surface, orients the antibody parallel to the membrane, where engagement of FcγR may be more restricted, and the HuB6 binding epitope makes the interaction with FcγR mild, potentially between the interactions of urelumab and utomilumab. Furthermore, IgG4 is known to engage FcγRIA and FcγRIIB more than IgG2, although both isotypes are generally characterized by relatively low FcγR interactions [16]. Our results also showed that FcγRs, especially FcγRIA, should be critical for the activity of HuB6, which also ensures its high safety. In contrast, urelumab is currently the strongest agonist and can even induce 4-1BB stimulation in the absence of FcγRs, and the IgG4 isotype likely boosts its in vivo activity with FcγR interaction, which may explain why it has severe hepatotoxicity. As an IgG2 mAb, Utomilumab has weak binding to FcγRIIA and FcγRIIB and does not even activate T cells in the presence of only FcγRIA, which may result in its weak activity. These data suggested that balancing agonistic activity with the affinities of FcγRs should be a strategy to screen 4-1BB agonistic mAb, and provided new evidence in understanding how therapeutic mAb works and new insight for the design of mAb candidate therapeutics.

Several important limitations should be considered in our study. First, the antitumor efficacy of HuB6 is mainly studied on CRC, which is also one of routinely selected types for cancer immunotherapy [31, 32], but more cancer types should be tested to explore the indication of HuB6 therapy. In addition, the capacity of HuB6, such as lymphocyte recruitment and activation in tumor microenvironment, needs to be determined in the future. Currently, HuB6 has been approved for evaluation in clinical trials based on the preclinical evidence, and multiple types of solid cancers including CRC will be investigated.

## Conclusions

We generated a novel humanized anti-4-1BB mAb, HuB6, that was shown to exert agonistic activity in tumors without systemic toxicity. HuB6 monotherapy demonstrated potent antitumor efficacy against CRC without systemic immune activation and had an enhanced tumor inhibitory effect when administered in combination with an anti-PDL1 antibody. These results strongly support the translation of HuB6 into clinical testing for the treatment of CRC and other solid tumors.

## Supplementary Information

**Additional file 1: Fig. S1** The interaction model between 4-1BB and HuB6 Fab. One copy of the complex is composed of chain A and chain E and F. Chain D interacts with chain B’ and C’ in another asymmetric unit form another copy with the same mode as chain B and C. 4-1BB is in blue and orange, HuB6 Fab H chain is in red, green, cyan and magenta. 4-1BB and HuB6 Fab in another asymmetric unit are in grey. **Fig. S2** The dependence on FcγR of the 4-1BB agonist activity. CD8 + T cells were cocultured with CHO-K1 cells expressing different FcγRs and treated with HuB6, utomilumab, urelumab or IgG control for 3 days in a CO2 incubator at 37 °C. After incubation, the secreted IFN-γ and IL-2 levels in the cell supernatants were determined by ELISA. **Fig. S3** Tumor photos of the MC38 and CT26 model mice. **a** the tumors of MC38 and CT26 model mice treated with the three doses of HuB6. **b** the tumors of MC38 and CT26 model mice treated with the three 4-1BB agonistic mAbs. **Fig. S4** Hematoxylin and eosin staining for major organs of the mice treated with HuB6. Major organs included heart, liver, lung, kidney and spleen.

## Data Availability

This article includes the datasets that support our findings.

## Abbreviations

ADCC: Antibody-dependent cell-mediated cytotoxicity
BLI: Biolayer interferometry
CDC: Complement-dependent cytotoxicity
CFSE: Carboxyfluorescein succinimidyl ester
CRC: Colorectal cancer
CRD: Cysteine-rich domain
ECD: Extracellular domain
EC50: Half-maximal effective dose
ELISA: Enzyme-linked immunosorbent assay
Fab: Antigen-binding fragment
Fc: Crystallizable fragment
HE: Hematoxylin and eosin
mAb: Monoclonal antibody
NK: Natural killer cell
PBMC: Peripheral blood mononuclear cell
PD-1: Programmed cell death-1
PD-L1: Programmed cell death ligand 1
scFv: Single-chain variable fragment
SPR: Surface plasmon resonance
TCR: T cell receptor
TNFRSF9: Tumor necrosis factor receptor superfamily member 9
