# Supplementary figures and images for: A humanized 4-1BB-targeting agonistic antibody exerts potent antitumor activity in colorectal cancer without systemic toxicity
Source: J Transl Med. 2022 Sep 8;20:415. doi: 10.1186/s12967-022-03619-w (PMC9461191; doi:10.1186/s12967-022-03619-w)

**Figure S1**


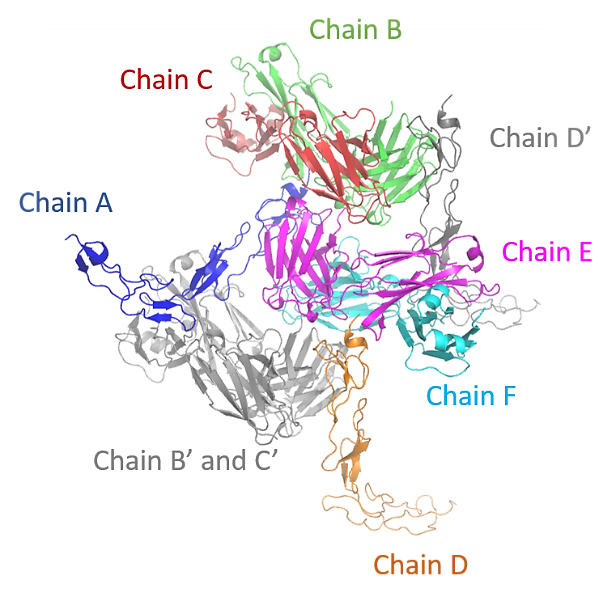


**Figure S2**


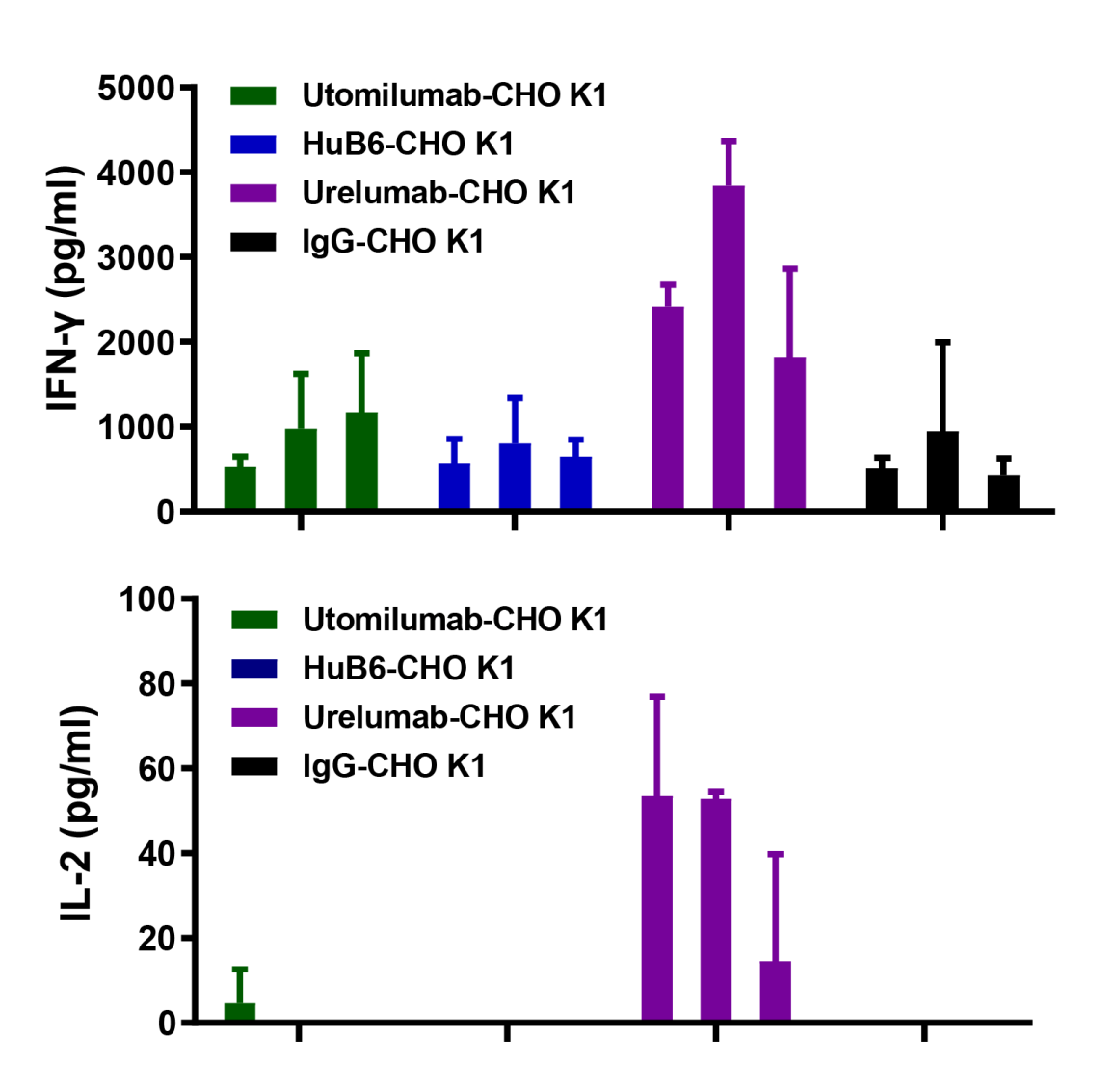


**Figure S3**


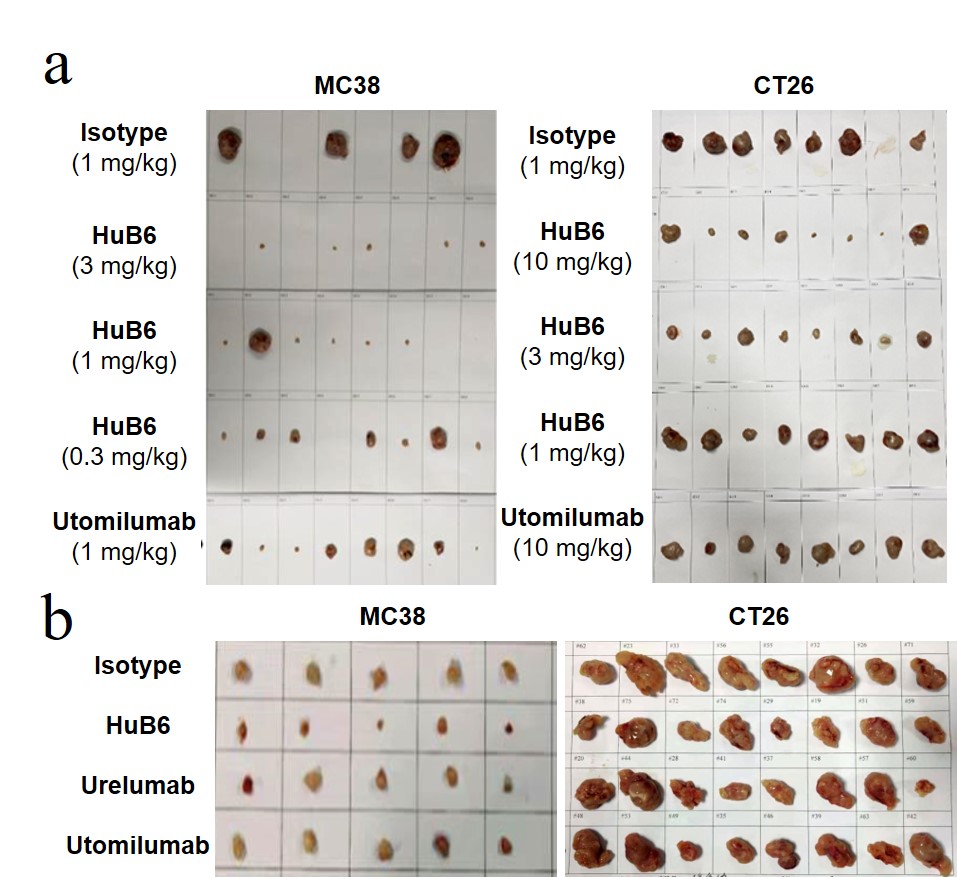


**Figure S4**


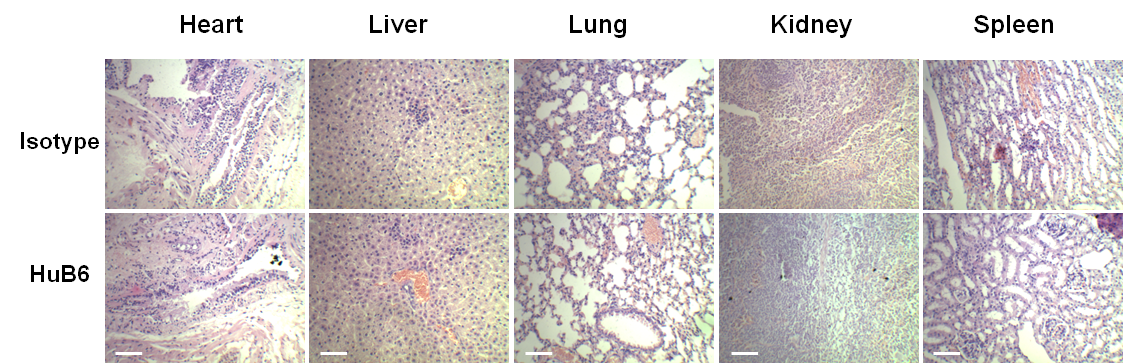

Supplement: Supplementary file 1 — Additional file 1: Fig. S1 The interaction model between 4-1BB and HuB6 Fab. One copy of the complex is composed of chain A and chain E and F. Chain D interacts with chain B’ and C’ in another asymmetric unit form another copy with the same mode as chain B and C. 4-1BB is in blue and orange, HuB6 Fab H chain is in red, green, cyan and magenta. 4-1BB and HuB6 Fab in another asymmetric unit are in grey. Fig. S2 The dependence on FcγR of the 4-1BB agonist activity. CD8 + T cells were cocultured with CHO-K1 cells expressing different FcγRs and treated with HuB6, utomilumab, urelumab or IgG control for 3 days in a CO2 incubator at 37 °C. After incubation, the secreted IFN-γ and IL-2 levels in the cell supernatants were determined by ELISA. Fig. S3 Tumor photos of the MC38 and CT26 model mice. a the tumors of MC38 and CT26 model mice treated with the three doses of HuB6. b the tumors of MC38 and CT26 model mice treated with the three 4-1BB agonistic mAbs. Fig. S4 Hematoxylin and eosin staining for major organs of the mice treated with HuB6. Major organs included heart, liver, lung, kidney and spleen. [file 12967_2022_3619_MOESM1_ESM.docx]
